# Pleural Mesothelioma: A Rapid Evolution of an Indolent Disease

**DOI:** 10.7759/cureus.33965

**Published:** 2023-01-19

**Authors:** Miguel Romano, Pedro Pinto, Raquel Afonso, Joana Fontes, Manuel Ferreira

**Affiliations:** 1 Internal Medicine, School of Medicine, Minho University, Braga, PRT; 2 Internal Medicine, Unidade Local De Saude Do Alto Minho, Viana do Castelo, PRT; 3 Internal Medicine, Unidade Local de Saude do Alto Minho, Viana do Castelo, PRT; 4 Internal Medicine, Hospital de Santa Luzia Viana do Castelo, Viana do Castelo, PRT; 5 Serviço de Medicina Interna, Hospital Conde de Bertiandos, Viana do Castelo, Viana do Castelo, PRT

**Keywords:** treatment, prognosis, diagnosis, pneumology, oncology, malignant pleural mesothelioma (mpm), epithelioid mesothelioma

## Abstract

Mesothelioma is a rare and insidious neoplasm and is characterized by its highly malignant and aggressive nature. The most common etiology is asbestos exposure, but there are some reports without known asbestos exposure and other factors leading to malignant pleural mesothelioma (MPM).

Here, we present the case of a 58-year-old woman with pleuritic chest pain, dyspnea, and fever on presentation to the emergency department (ED), which caused several admissions to the ED in 20 days. The patient was then admitted to the internal medicine department with a diagnosis of community-acquired pneumonia with parapneumonic effusion. During hospitalization, a positron emission tomography (PET) scan, thoracic computed tomography (CT), and pleural biopsy were performed and a final diagnosis of malignant epithelioid pleural mesothelioma was made. Six weeks after the onset of symptoms, the patient presented with an exponential disease progression, dying two months after the diagnosis, despite the initiation of chemotherapy. MPM remains a diagnostic and therapeutic challenge with a very poor prognosis. However, studies show that mesothelioma patients who undergo treatment live at least twice as long as patients who do not receive treatment. This case report is particularly significant because, although it was epithelioid mesothelioma, multiple solid masses were noted on CT and the patient exhibited rapid disease progression, dying a few weeks after starting treatment.

## Introduction

Mesothelioma is a rare neoplasm with an estimated incidence of about 20 per million inhabitants in Europe and it is increasing worldwide [1]. It is known to be insidious due to its long latency period which can last up to 40 years [2]. More frequently, it arises from the mesothelial tissue surfaces of the pleura but can also occur in the peritoneum and tunica vaginalis [3]. Malignant pleural mesothelioma (MPM) remains a severe problem as the incidence continues to increase worldwide [4] and is likely to peak until 2030 [5]. MPM occurs predominantly in men (with a male-to-female ratio of 5:1), and the risk increases with age [6], with the vast majority occurring in patients aged 60 years or older [7]. Asbestos exposure is the most commonly known etiology linked to mesothelioma, however, there are some case reports where no exposure has been found, and some other factors have been emerging as important causes of MPM [8-10].

The clinical manifestations of MPM patients vary widely, with thoracodynia, dyspnea, and pleural effusion being typical symptoms, often accompanied by weight loss, weakness, and other symptoms. The onset of symptoms is known to be insidious and nonspecific, leading to a high rate of misdiagnosis [11]. Pleural biopsy is the gold standard for the definitive diagnosis of MPM, however, some authors stated that it is difficult to make a definite diagnosis by pleural biopsy alone and that the accuracy of the diagnosis could be improved by adding immunohistochemical examination [12].

MPM is characterized by its high malignancy and aggressiveness and patients, if left untreated, present a median survival time of only four to 12 months [13]. There are various treatment options, but comprehensive treatment with resection of the visible tumor in combination with radiotherapy, chemotherapy, and immunotherapy has been the most promising strategy to date [14,15]. Non-randomized studies in carefully selected patients reported median survival times of up to 29 months and a systematic review concluded that trimodality treatment may be beneficial for certain patients [16]. Nevertheless, there are several new treatments under investigation, and the future of MPM seems to include many more options than the ones available now, evolving into a highly individual and personalized treatment. 

## Case presentation

We present the case of a 58-year-old woman with a history of endometroid adenocarcinoma, which was treated by surgery and followed up by chemoradiotherapy in February 2021. On May 2021, the patient was admitted to the emergency department (ER) with pleuritic chest pain, dyspnea, and fever.

**Figure 1:**
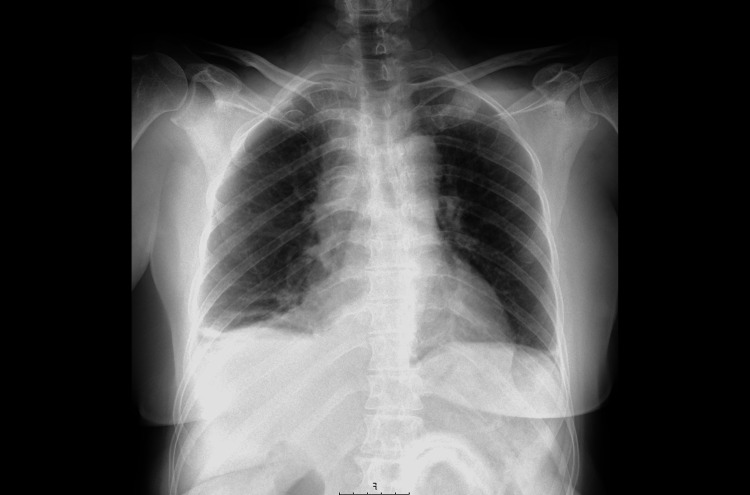
Thoracic X-ray at first ED admission Thoracic radiography with no evident lesions but with a very small pleural effusion on the right side

The chest radiography (Figure 1) showed a very small and free pleural effusion on the right side. The patient was discharged home with a prescription for antibiotics for suspected right-sided community-acquired pneumonia. Over the next 10 days, the patient was admitted to ER three more times for worsening symptoms and received three cycles of various antibiotic therapies. However, the patient's dyspnea and orthopnea continued to gradually worsen.

**Figure 2:**
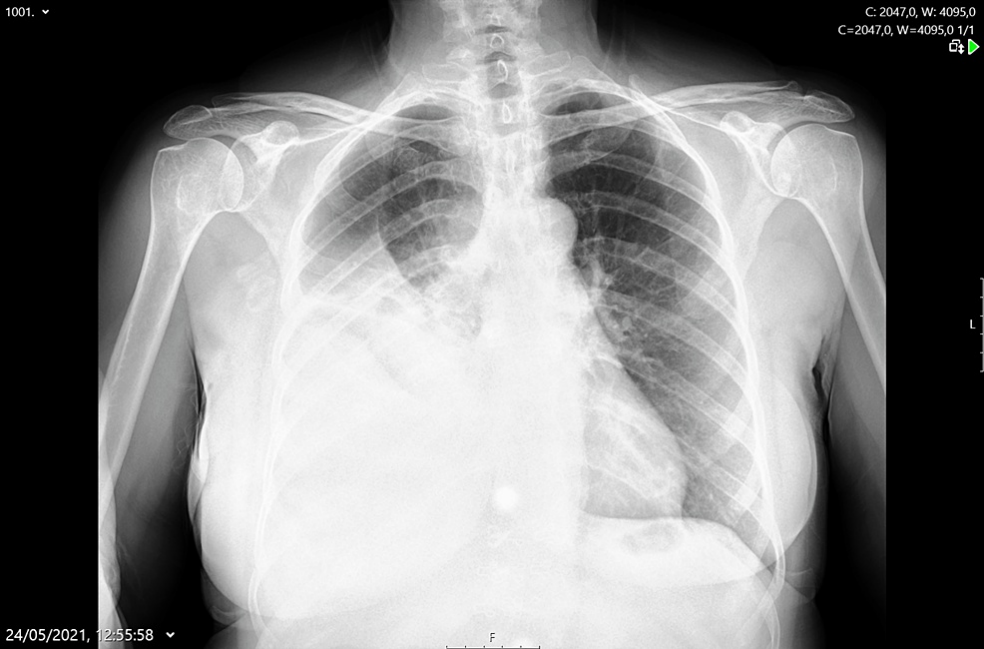
Thoracic X-ray at internal medicine admission Thoracic radiography with medium-sized right-side pleural effusion and disease progression

One month after the first episode, on another admission to the ER, the thoracic radiography (Figure 2) showed a medium-sized pleural effusion on the right side, and a thoracentesis was performed, which revealed a yellow effusion with exudate characteristics. The patient was then admitted to the department of internal medicine with a diagnosis of right-sided pneumonia with accompanying pleural effusion, and piperacillin-tazobactam was prescribed. During hospitalization, given the pathological neoplastic history, the case was discussed with the gynecology department, and it was decided to perform a Positron Emission Tomography (PET) scan.

**Figure 3:**
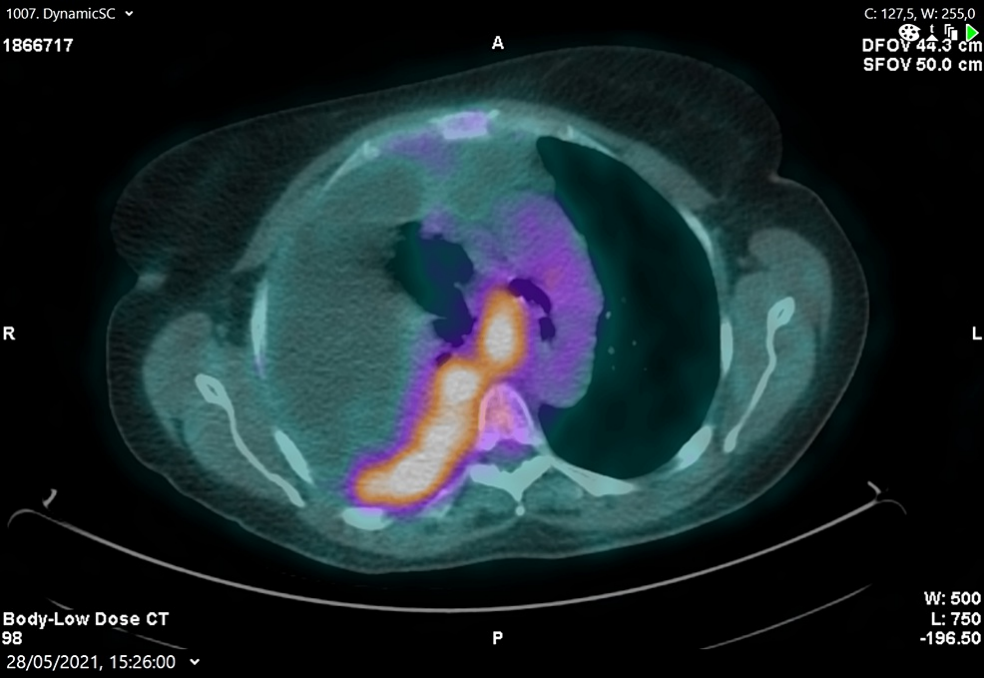
PET scan showing right-side pleural involvement

PET scan (Figure 3) showed extensive malignant involvement of the pleura with pleural effusion, with no evidence of other lesions elsewhere, and the diagnosis of endometrioid adenocarcinoma metastasis became less likely and the hypothesis of a primary pulmonary lesion came to the fore.

**Figure 4:**
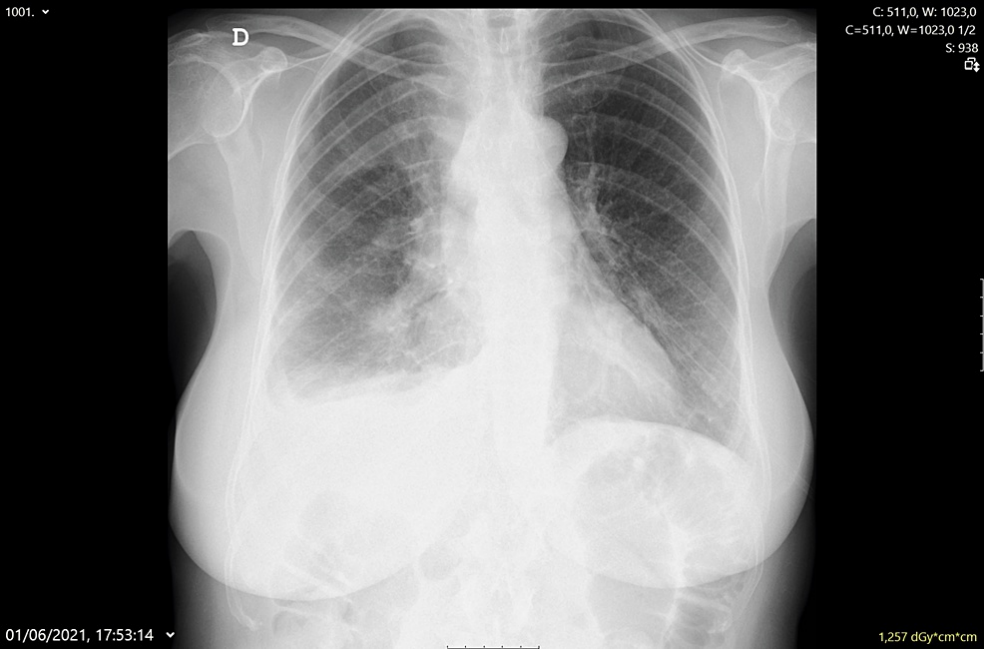
Thoracic X-ray after thoracocentesis

The case was then also discussed with the pulmonology department, and a pleural biopsy and a therapeutic thoracentesis were performed (Figure 4). Two weeks after hospitalization, the patient presented with clinical deterioration.

**Figure 5:**
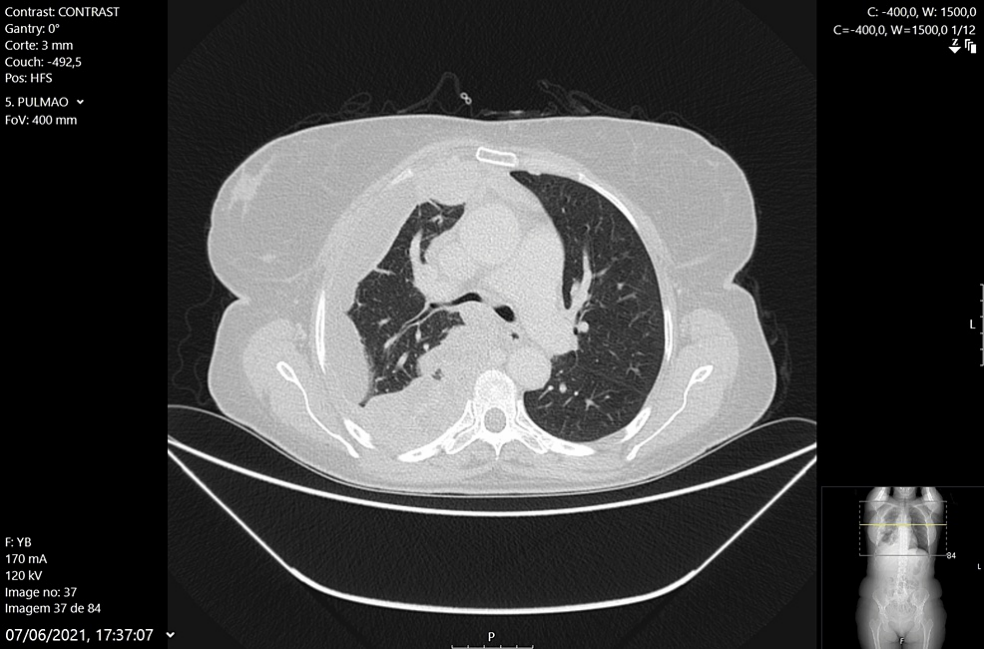
Thoracic CT scan during hospitalization showing rapid progression

Thoracic CT was repeated (Figure 5) and showed rapid progression with the appearance of multiple solid masses in the pleura and right pleural space with a small localized pleural effusion, supporting the hypothesis of mesothelioma. The result of the pleural biopsy confirmed the diagnosis of epithelioid mesothelioma. Regarding the diagnosis, it is important to notice that the patient denied any known exposure to asbestos, including occupational exposure.

**Figure 6:**
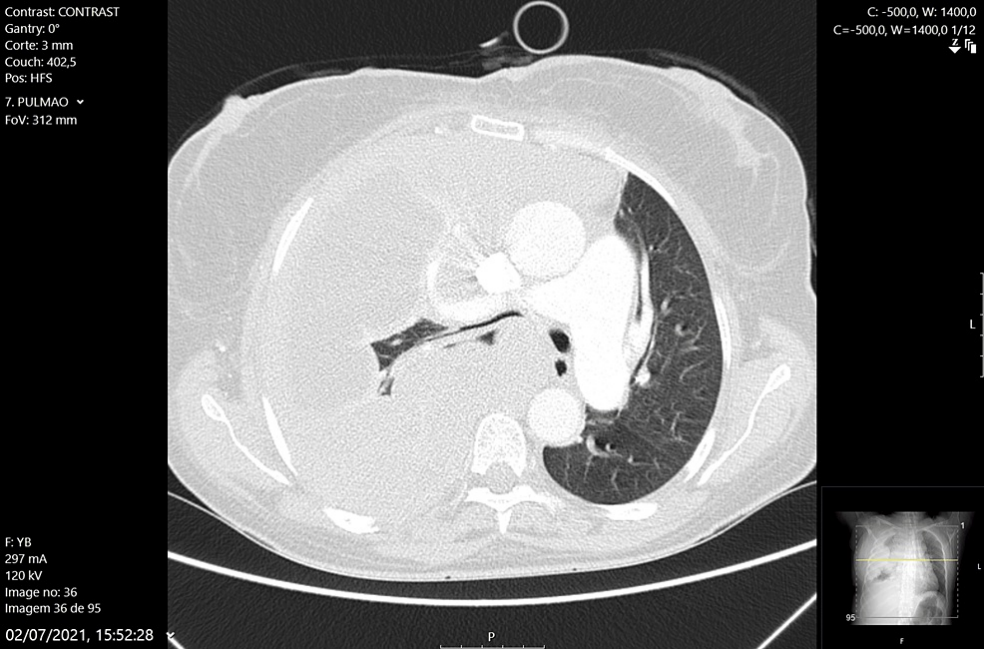
Thoracic CT scan showing almost full involvement on the right side

The patient was then referred to the pulmonology-oncology department, where she underwent two cycles of chemotherapy. However, she showed exponential progression of the disease (Figure 6) and died two months after diagnosis.

## Discussion

MPM is a complex disease that causes significant morbidity and mortality, and its actual global burden is still unclear. However, the disease has been widely recognized in asbestos-exposed populations since the 1960s, and the World Health Organisation Mortality Database estimates 38,400 mesothelioma deaths per year worldwide [17]. The prognosis of MPM is very poor and some population-based studies have identified independent risk factors for poor outcomes as non-epithelioid histology, increasing age, and male gender [18].

Maintaining a high index of suspicion is key to achieving an earlier diagnosis and, probably, a more successful treatment outcome. Nevertheless, to date, there are no standardized treatment strategies, and all of the therapies available are only expected to prolong survival time and improve the quality of life [19]. Three treatment approaches have evolved over the last decades: chemotherapy, radiotherapy, and surgery. Due to the failure of any single modality of treatment to significantly affect long-term survival, multimodality treatment including chemotherapy and radiotherapy has been proposed, even though the exact sequence of treatment is yet to be found as the relevant clinical trials have not been conducted. Treatment options remain limited, but the future seems to include newer therapies and personalized and individual approaches. Novel agents are being studied, and targeted therapies to epidermal growth factor receptor antagonists and platelet-derived growth factor receptor inhibitors, immunotherapy such as tremelimumab and pembrolizumab, and mesothelin-targeted treatments are examples of some of the several investigation areas on development regarding mesothelioma treatment [16].

Here we present a case of MPM in which wasn't found any etiologic association, including asbestos exposure. Another point of particular importance in this case is the unusually severe course of the disease despite the absence of the main identified risk factors. Regardless of the histologic diagnosis of epithelioid mesothelioma, multiple solid masses were found, and the patient had rapid disease progression and died within a few weeks after starting chemotherapy. This range of presentation and evolution emphasizes the difficulty of achieving an early diagnosis of this type of neoplasm.

## Conclusions

MPM remains a diagnostic and therapeutic problem because of long latency, the need for histologic confirmation, limited therapeutic options, and poor prognosis. In developed countries, residual asbestos exposure is a continuing risk that will most likely lead to a mesothelioma epidemic in the future. Nevertheless, treatment is the best way to improve the prognosis of mesothelioma, which may mean an improvement in quality of life and an increase in life expectancy. Some studies show that mesothelioma patients who undergo treatment live at least twice as long as those who do not receive treatment. As with any rare disease, referral to experienced centers is recommended. This clinical case illustrates the diagnostic difficulties that are quite common in mesothelioma and should warn the medical community to be aware of its projected increase in incidence by 2030.
